# Process Understanding of Plasma Electrolytic Polishing through Multiphysics Simulation and Inline Metrology

**DOI:** 10.3390/mi10030214

**Published:** 2019-03-26

**Authors:** Igor Danilov, Matthias Hackert-Oschätzchen, Mike Zinecker, Gunnar Meichsner, Jan Edelmann, Andreas Schubert

**Affiliations:** 1 Professorship Micromanufacturing Technology, Faculty of Mechanical Engineering, Chemnitz University of Technology, 09107 Chemnitz, Germany; matthias.hackert@mb.tu-chemnitz.de (M.H.-O.); mike.zinecker@mb.tu-chemnitz.de (M.Z.); andreas.schubert@mb.tu-chemnitz.de (A.S.); 2 Fraunhofer Institute for Machine Tools and Forming Technology, 09126 Chemnitz, Germany; Gunnar.Meichsner@iwu.fraunhofer.de (G.M.); jan.edelmann@iwu.fraunhofer.de (J.E.)

**Keywords:** plasma-electrolytic polishing, PeP, surface modification, finishing, electro chemical machining, ECM

## Abstract

Currently, the demand for surface treatment methods like plasma electrolytic polishing (PeP)—a special case of electrochemical machining—is increasing. This paper provides a literature review on the fundamental mechanisms of the plasma electrolytic polishing process and discusses simulated and experimental results. The simulation shows and describes a modelling approach of the polishing effect during the PeP process. Based on the simulation results, it can be assumed that PeP can be simulated as an electrochemical machining process and that the simulation can be used for roughness and processing time predictions. The simulation results exhibit correlations with the experimentally-achieved approximation for roughness decrease. The experimental part demonstrates the results of the PeP processing for different times. The results for different types of roughness show that roughness decreases exponentially. Additionally, a current efficiency calculation was made. Based on the experimental results, it can be assumed that PeP is a special electrochemical machining process with low passivation.

## 1. Introduction and Literature Review

### 1.1. Introduction

**Figure 1:**
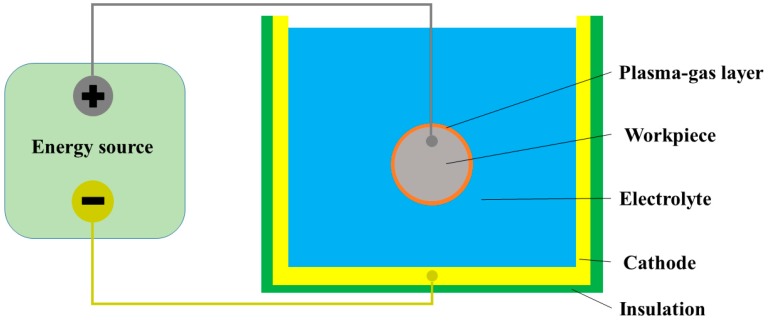
Principle scheme of PeP.

Plasma electrolytic polishing (PeP) is a special case of electrochemical machining [1] which requires high voltage and uses an environmentally-friendly aqueous solutions of salts. A summary of the PeP process is shown in Figure 1, where the workpiece is an anode connected to a DC energy source.

PeP is a technology that is used as a finishing surface treatment for precision metallic parts because of low achievable roughness (*Ra* < 0.02 μm) and low removal rates [2,3]. Nowadays state-of-the-art method for polishing is mechanical polishing. In comparison to mechanical polishing, in PeP, the whole workpiece surface can be polished in a few minutes, as can complex free forms [2,4,5].

In the extant literature, there is a lot of information on solutions used for polishing different metal alloys (e.g., steels, aluminium, titan and others) [5,6,7,8,9,10,11,12] and process parameters (e.g., temperature, electrolyte concentration, voltage, etc.). For example, 3–6% ammonium sulphate solutions are widely used for polishing steel workpieces; the applied voltage is between 200 V and 350 V; a combination of ammonium fluoride and potassium fluoride is common for polishing titanium parts, etc. For metals, the common temperature range is 70–90 °C [5,10,12].

**Figure 2:**
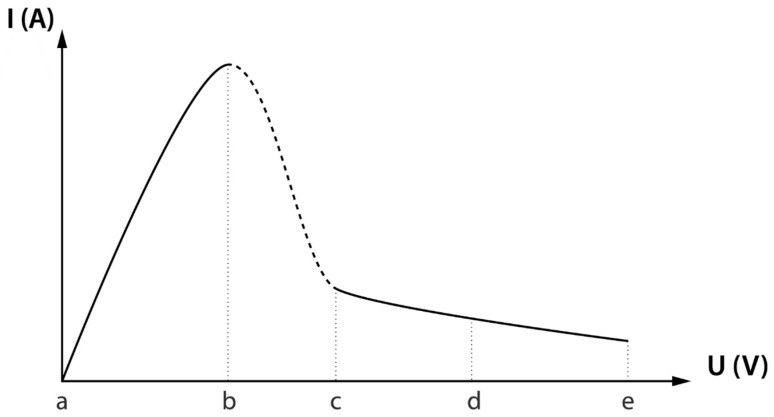
Schematic current–voltage characteristic according to [5].

Figure 2 shows a typical current vs. voltage plot. The first section, “ab”, is a conventional electrolysis process that can be described by classical electrochemistry [5]. Section “bc” is a transient or switching mode, when a plasma-gas layer periodically occurs on the anode [5]. The section “cd” is an electrolytic plasma mode [6] when the plasma-gas layer is stable and polishing is possible. At section “de”, the plasma-gas layer becomes unstable. At sections “bc”, “cd” and “de”, an increase in voltage leads to a decrease in current because of the increase in the thickness of the plasma-gas layer [13].

However, the minimum achievable roughness is limited by the initial roughness of the workpiece. Mukaeva [14] has shown that the surface roughness parameter Ra can be approximated by the following parametric dependence on time t:

(1) Ra=A⋅exp(−t/τ)+C

where:

A – max decrease in roughness;
τ – time constant;
C – min achievable roughness;
t – processing time;

All of these variables can be obtained with experimental data. 

**Figure 3:**
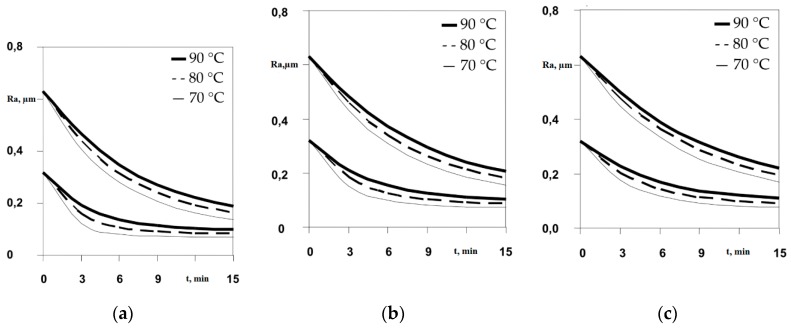
Roughness as function of time, (**a**) 250 V, (**b**) 300V, (**c**) 350 V [14].

The parameters used by Mukaeva for the experiments are provided in Table 1. The results are shown in Figure 3. The roughness parameter Ra is plotted as a function of time for three different voltage values: 250 V, 300 V, 350 V. From Figure 3, it can be seen that with higher temperatures, the achievable roughness is greater; this can be explained as follows. Higher temperatures increase the thickness of the plasma-gas layer, leading to a decrease in current [3,13].

Some authors have demonstrated that surface roughness depends on the immersion depth of the sample: the deeper the sample was immersed, the lower the achieved surface roughness [3,15,16]. This effect can be explained by the fact that the plasma-gas layer is not uniform along the workpiece height. The thickness of the plasma-gas layer closer to the electrolyte-air interface is higher [15,16]. This happens because the gas bubbles move towards the electrolyte-air interface.

Some information about PeP and workpiece geometry was provided by Kulikov et al. [5]. Their experiments show that products with small cavities, when the depth is less than the diameter of the bore, can be polished. Inside the deep bores, the walls were not polished when the depth exceeds the bore diameter. Nevertheless, a large number of deep bores, cavities and cracks leads to an increase in the current, and also can lead to interruptions in the polishing process. 

A possible solution for the problem with polishing difficult geometries, small bores and cavities is the usage of an electrolyte jet [4,5,17,18,19,20,21,22]. Ablyaz et al. [17] and Novoselov et al. [22] demonstrated the possibility of polishing an object with a complex geometry with a free electrolyte jet. In the works of Alekseev et al. [20] and Cornelsen et al. [4], the polishing of the inner surfaces of pipes was shown. 

Regarding the process, there are still two big gaps in our understanding of the PeP process: the detailed description of conductivity of the plasma-gas layer and the polishing mechanism of PeP. The information provided below is an overview of existing theories on the process.

### 1.2. Electrical Conductivity of the Plasma-Gas Layer

There are several theories of the nature of the electrical conductivity of the plasma-gas layer. One of the first to have observed the plasma-gas layer within the anode process at the high voltages was Kellogg [23]. In his work, Kellogg hypothesised that the plasma-gas layer is “primarily a water-vapour film”. He observed some sparks inside plasma-gas layer during the process and assumed that conduction could be explained by the ionisation of the gas within the plasma-gas layer, caused by a high electric field. 

A lot of papers have examined this of or a similar mechanism of conduction [10,14,16,24,25,26]. A high electric field causes ionisation on the vapour film that surrounds workpiece, and the formation of plasma. So, the plasma-gas layer consists of ions from the electrolyte and ions from the workpiece surface. In some papers, ions from the electrolyte and metal ions play the main role in the conduction of the plasma-gas layer [10,16,25].

Others assume that ions have an important, but not a principal, role in conduction. According to them, the values of current densities, which were observed in experiments, cannot be provided only by ions. In this work, ions contribute to the release of electrons that provide the necessary conductivity [14,24,26].

Vaňa et al. [27] mentioned that the plasma-gas layer is mainly water steam. The water steam layer is ionized due to high voltage. This leads to the formation of electric current which flows in the form of a glow discharge.

**Figure 4:**
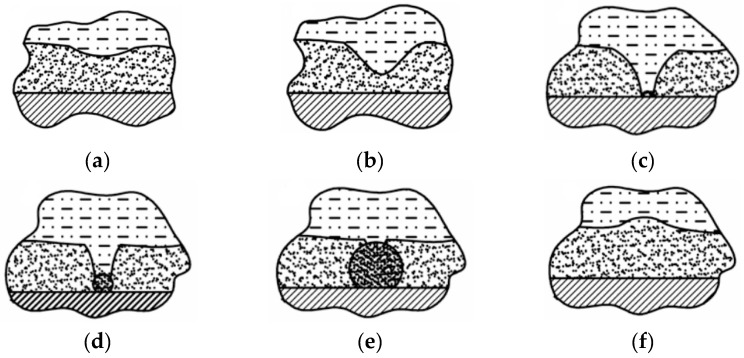
Electrolyte bridge formation scheme [28].

Another theory consists of the appearance of so-called electrolyte bridges [3,28,29]. In this theory, the thickness of the plasma-gas layer is not constant. The high electric field and the fact that the plasma-gas layer is not homogeneous lead to the appearance of zones with small thicknesses of the plasma-gas layer (Figure 4a). Then, under the ponderomotive force, the electrolyte continues to shift toward the workpiece surface, so the thickness of the plasma-gas layer gets smaller (Figure 4b). Getting closer to the workpiece surface, the electric field is higher, so the ponderomotive force is also higher. When the distance between the electrolyte and the surface of workpiece is around a couple of microns, additional, small “electrolyte bridges” appear (Figure 4c). When all this small bridges touch the surface, they create a current impulse and quickly boil because of Joule heating (Figure 4d,e). Sinkevitch et al. [3,28] compare this with explosive boiling. In this theory, the whole current in the system is a superposition of a large number of impulses from “electrolyte bridges”. Also, because of the small explosions of the bridges, the plasma-gas layer vibrates during the process. Sinkevitch also mentions that the current in the PeP process is a combination of a constant and a high-frequency component [3,28]. Duradji observed two components in the current during the polishing process [26]. 

**Figure 5:**
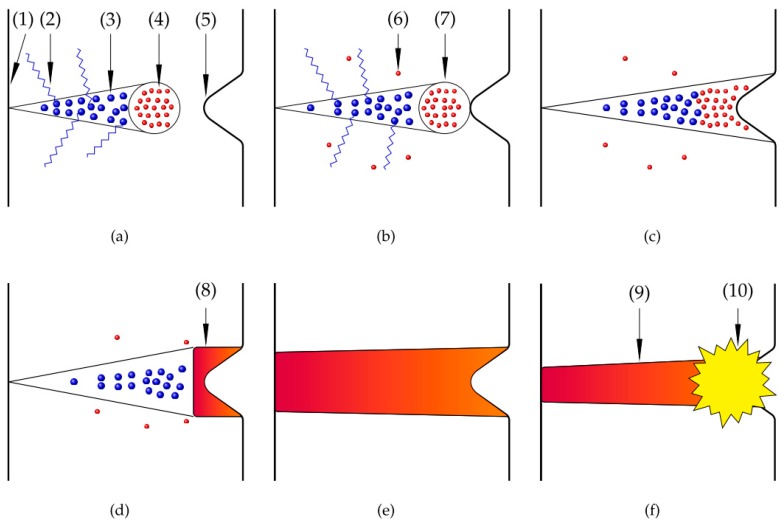
Schematic drawing of the formation of a plasma discharge according to streamer theory [4,30].

Another theory, called the ‘streamer theory’, is based on streamer discharges [4,30,31]. A schematic drawing is provided in Figure 5. In this figure (1) is electrolyte, (2) photoionization, (3) ions, (4) electrons, (5) workpiece surface, (6) secondary electrons, (7) avalanche head, (8) the streamer, (9) plasma channel, and (10) a gas explosion. Because of the high electric field, electrons have enough speed and energy to ionize molecules and release other electrons from them (Figure 5a). These electrons strike other molecules, forming some form of electron avalanches (Figure 5b). Then, the flow of electrons and ions forms a conductive channel that connects the workpiece surface with the electrolyte (Figure 5c–f)). 

In summary, none of the presented models fully describes the conduction in plasma-gas layer.

### 1.3. Mechanism during Plasma Electrolytic Polishing

In the literature, there are many theories about the mechanisms of polishing during the PeP process. Many papers mention melting the workpiece surface as the main, or as an important, part of the polishing mechanism [4,5,14,25,27,29,30,31,32].

In the streamer theory, mentioned above, melting is considered a primary polishing mechanism [4,30,31,32]. In this theory, ions and electrons move toward the workpiece surface through a conductive channel, i.e., a streamer, and start to interact with the workpiece. This interaction leads to an increase in temperature of both the workpiece surface and the conductive channel, and melting. A further increase in temperature leads to an explosion in the channel. The explosion removes the melted metal. 

Plotnikov et al. [25] suggested a mechanism which is a combination of melting and oxidation. At the beginning, a new gas bubble is formed; this is caused by the high temperature of the workpiece. Because of the high electric field, the gas inside the bubble gets ionized and turns into a high temperature plasma which starts to melt the oxide layer on the workpiece surface. The plasma bubble expands due to the high temperature, thereby creating a shock wave. The shock wave partially reflects from the interface between the plasma-gas layer and the electrolyte. The reflected wave compresses the gas bubble, causing it to collapse. When the bubble collapses, a process similar to cavitation occurs. Ions get into the formed void. Then, ions start to react with the workpiece surface, leading to the formation of an oxide film. According to this model, the polishing process takes place when the rate of the oxide layer formation is comparable to the rate of its removal by high-temperature plasma. Gas bubbles are present on the entire workpiece surface, so the polishing takes place everywhere. However, the removal rate for the peaks is much greater than that for the cavities.

**Figure 6:**
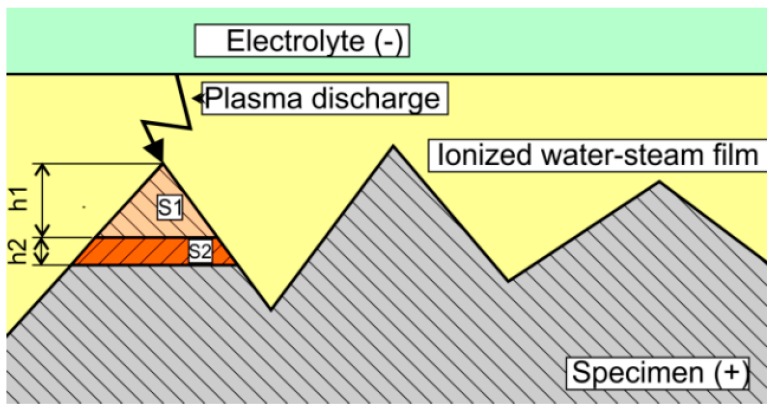
Schematic representation of the polishing process according to Vaňa et al. [27].

Vaňa et al. [27] mentioned that the plasma-gas layer is an ionized medium in which glow discharges are present.. Discharges melt the workpiece surface first at the points where the thickness of the plasma-gas layer is lower. This leads to the rapid removal of the peaks on the workpiece surface, and to a smoothing effect. Because each plasma discharge removes the same amount of material (S1 = S2), the thickness of removed layer h2 is less than that of h1. According to Vaňa, this leads to a slow down of the removal rate over time. A schematic representation of the polishing process can be seen in Figure 6. However, it should be noted that better gloss can be achieved only by using the proper electrolyte. This may indicate the important role of electrochemical reactions in the polishing process.

Another approach considers PeP as an electrochemical process [3,5,10,29,33,34,35,36,37].

**Figure 7:**
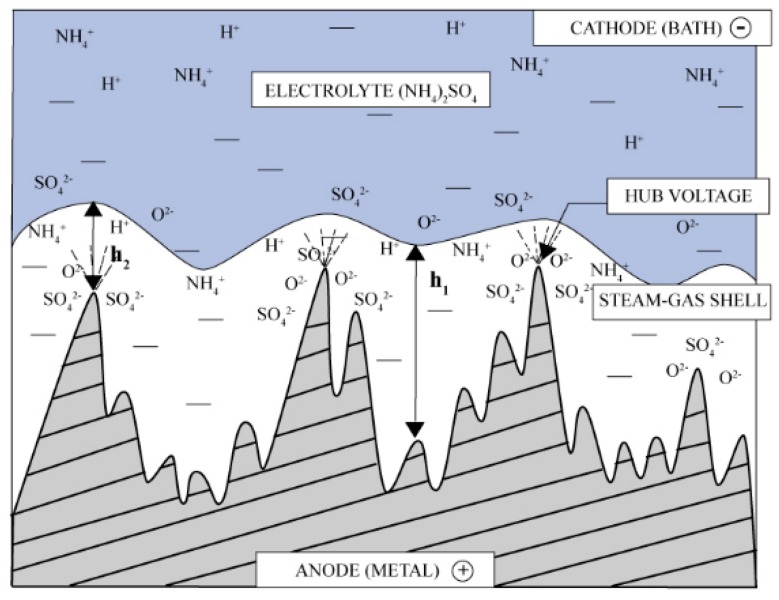
The mechanism of electrolytic-plasma polishing according to Kalenchukova et al. [10].

According to Kalenchukova et al. [10], there is no melting during the PeP process; the main polishing mechanism is electrochemical dissolution. The plasma-gas layer (named steam-gas shell in Figure 7) has a different thickness on the peaks (h2) and in the cavities (h1), leading to a higher current density on the peaks, and consequently, to a higher removal rate. Higher removal of the peaks leads to a rapid decrease in roughness. 

Smyslov et al. [36] describe the polishing mechanism as a superposition of anodizing and simultaneous chemical etching of the formed oxide layer. Etching the peaks is faster, and the oxide layer there is thinner than that in the cavities; this leads to a decrease in roughness. 

Parfenov et al. [34] have studied the current efficiency of the PeP and electrolysis processes. It was concluded that PeP is mainly an electrochemical process, with a current efficiency of about 30%. It was stated that there is no melting during the PeP process, despite the presence of discharges in the plasma-gas layer. These discharges do not cause melting or the removal of the workpiece surface. Based on data obtained from a comparison of PeP and electrolysis by Parfenov et al. [34], it can be concluded that the appearance of a plasma-gas layer leads to changes in some electrochemical reactions. By traditional electrolysis, the formation of oxygen can be observed on the anode surface. Electrochemical machining (ECM) with the same current density as in PeP process has a current efficiency of about 9% [34]. The low current efficiency of the ECM process can be explained by the passivation of the workpiece surface by oxygen. In the case of PeP, because of a stable plasma-gas layer, it can no longer be due to oxygen formation on the workpiece surface. It can therefore be assumed that oxygen formation still takes place, albeit on the interface between the plasma-gas layer and the electrolyte. A similar assumption was made by Kellogg [23].

Sinkevitch et al. [3,35,37] assumed that the PeP process can be considered as an anodic dissolution that can be described by the mechanism of complexation through a series of sequential or sequential and parallel stages.

Volenko et al. [29] assumed PeP as a superposition of physicochemical, thermal, electrical and hydrodynamic processes. According to Volenko, the plasma-gas layer is a dielectric. The conduction in the plasma-gas layer is described by the electrolytic bridges model, as mentioned above. In this paper, polishing is a combination of electrochemical removal and electrical discharge machining (EDM). The same mechanism is mentioned by Saushkin et al. [14]. 

Kulikov et al. [5] mentioned that the polishing mechanism is not fully understood, but assumed that polishing can occur because of a combination of electrochemical dissolution and melting by the discharges.

Alekseev et al. [16] describe the polishing mechanism as a combination of discharges, ion sputtering and chemical sputtering. 

## 2. Multiphysics Simulation of Plasma Electrolytic Polishing

**Figure 8:**
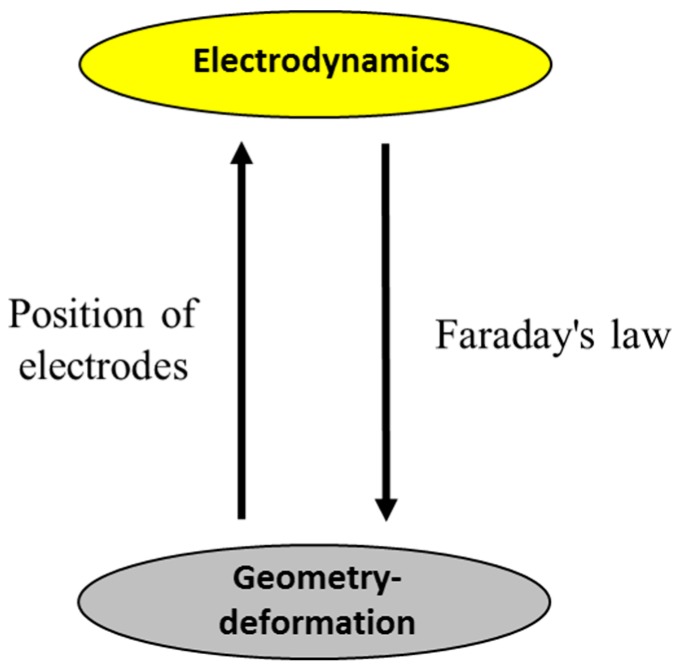
Coupling scheme of the multiphysical model [38].

To simulate the PeP process, a model was developed. Based on a literature review and on experimental experience, the simulation in this paper is based on the assumption that the main mechanism of PeP for stainless steel is electrochemical. The coupling scheme of the model is provided in Figure 8. The model set up and calculation were made in COMSOL Multiphysics^®^. Electric Currents and Deformed Geometry interfaces were chosen for this model to simulate the current and electric potential and the polishing effect during the PeP process. The developed model is used to simulate the PeP process after the appearance of a stable plasma-gas layer. 

### 2.1. Geometry 

**Figure 9:**
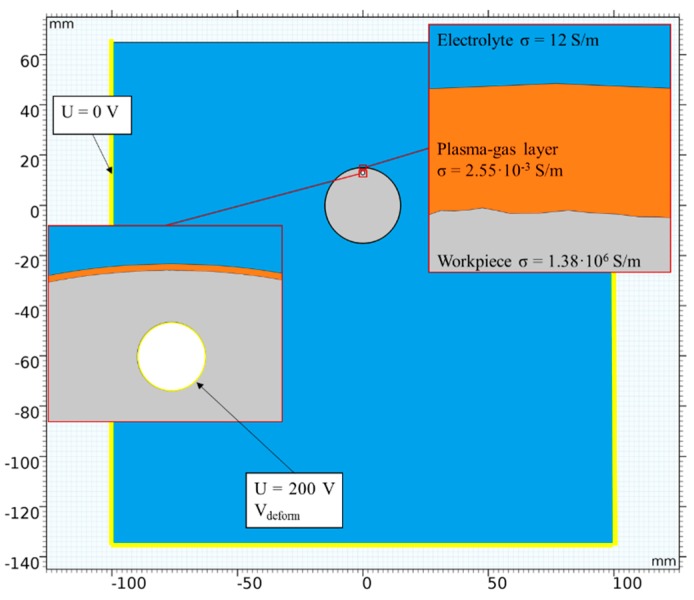
Modell geometry and boundary & domain conditions.

The model geometry and boundary conditions are provided in Figure 9. The model set up was based on the principle scheme shown in Figure 1. The bath with the electrolyte had dimensions of 20 cm × 20 cm. The workpiece was a disc with a mounting bore, which was completely immersed in the bath to a depth of 5 cm. A plasma-gas layer surrounded the workpiece from the beginning of the simulation.

The model had 3 domains: an electrolyte, plasma-gas layer and the workpiece. The side and bottom boundaries of the model were grounded. A voltage of 200 V was applied to the boundaries of the mounting bore. 

To simulate the polishing effect of PeP and to analyse the current density distribution on a surface, the workpiece surface profile was generated randomly in COMSOL Multiphysics^®^ using the Spatial Frequencies method [39] and the following equations:

(2) $$y=\sin\left(2\pi s\right)\left(15+A\overset{N}{\underset{m=-N}{\sum }}{\left(m^{2}\right)}^{\frac{-b}{2}}g1\left(m\right)\cos\left(2\pi ms+u1\left(m\right)\right)\right),$$

(3) $$x=\cos\left(2\pi s\right)\left(15+A\overset{N}{\underset{m=-N}{\sum }}{\left(m^{2}\right)}^{\frac{-b}{2}}g1\left(m\right)\cos\left(2\pi ms+u1\left(m\right)\right)\right),$$

The parameters that were used for this are provided in Table 2. The initial roughness in the model was 2.49 µm. Electrical conductivity of the electrolyte domain was set 120 mS/cm. This value corresponded to an ammonium sulphate solution with a concentration of 50 g/L at 75 °C [13]. This electrolyte is common for polishing stainless steels. Steel 1.4301 was chosen as the material for the anode.

Electrical conductivity of the plasma-gas layer was calculated based on the assumption that almost all voltage would drop in the plasma-gas layer. Using experimental data and data provided in the existing literature, it was possible to calculate first electric field, and then, the electrical conductivity. For this calculation, a thickness of 150 μm for the plasma-gas layer was chosen, based on the literature [2,3,4]. Then, based on the chosen thickness of the plasma-gas layer and a voltage of 200 V that is used in the model, the electric field can be calculated as follows:

(4) $$E=\frac{V}{dh}=\frac{200\;V}{0.015\;\mathrm{cm}}=13333\;V/\mathrm{cm}.$$

This corresponds with the range mentioned in extended literature; common values of the electric field are 10^4^–10^5^ V/cm [2,3,4,14]. The current density can be calculated with following equation: $j_{n}=\sigma \cdot E$. Using the current density and the electric field, the electrical conductivity can be calculated. Taking the average $j_{n}$ based on experimental data from Rajput et al. [13] for 200 V of 0.3399 A/cm^2^ and the above calculated electric field, the electrical conductivity of the plasma-gas layer can be calculated as follows:

(5) $$\sigma =\frac{j_{n}}{E}=\frac{0.3399\;A/{\mathrm{cm}}^{2}}{13333\;V/\mathrm{cm}}=2.55\cdot 10^{-2}\mathrm{mS}/\mathrm{cm}.$$

Other simulation parameters are provided in Table 3.

### 2.2. Model Mesh

**Figure 10:**
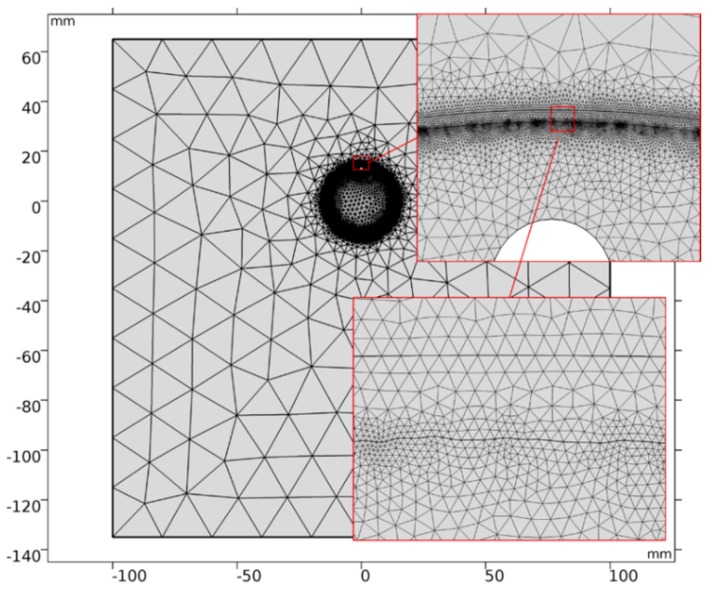
Visualisation of the model mesh.

A visualisation of the model mesh is provided in Figure 10. The complete mesh consists of 213,870 domain elements and 9247 boundary elements. All parameters used for meshing are provided in Table 4. The finest mesh is realised near the anode surface where the removal takes place.

The mesh deformation is calculated according to equation below:

(6) $$V_{\mathrm{deform}}=K\cdot \left(-j_{n}\right),$$

where:

K is the removal coefficient, and $j_{n}$ is the normal current density. K is calculated from experimental data from Rajput et al. [13] and based on the average removal speed in a one-dimensional direction and on the average current density. For example, the average removal speed and current density for 200 V can be used to determine the next removal coefficient:

(7) $$K=\frac{MRR}{j_{n}}=\frac{5.24\times 10^{-8}\;m/s}{3398.69\;A/m^{2}}=1.54\times 10^{-11}m^{3}/\left(A\cdot s\right),$$

**Figure 11:**
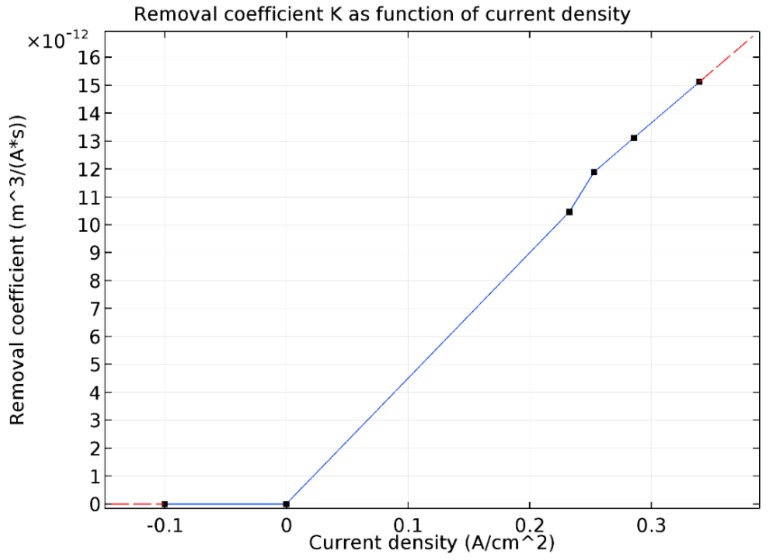
Removal coefficient K as function of current density.

It was assumed for this model that voltage would only have an influence on the thickness of the plasma-gas layer. In this case, it may be concluded that the MRR is primarily dependent upon the current density. So, the removal coefficient K can be described as a function of the current density. The removal coefficient K as a function of current density can be seen in Figure 11. The black dots on the graph represent data obtained from experimental data from Rajput et al. [13]. It was assumed that for a current density equal to and less than zero, a metal removal process does not occur. A linear approximation was also applied for current density values exceeding 0.34 A/cm^2^. A removal simulation was made for 300 s machining time.

### 2.3. Simulation Results

**Figure 12:**
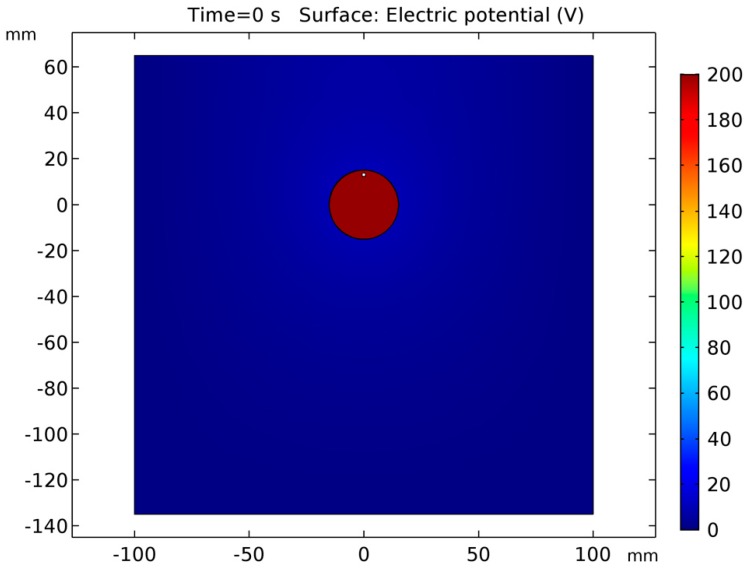
Electric potential.

**Figure 13:**
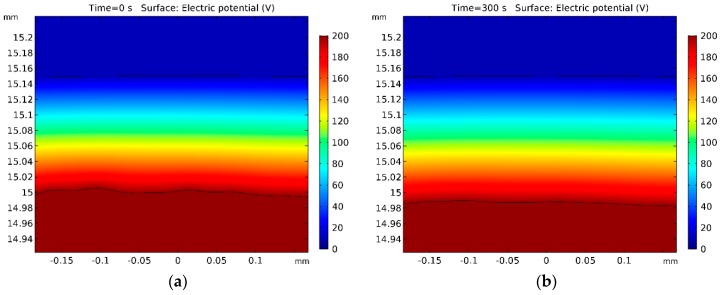
Detailed view of workpiece surface and electric potential (**a**) at t=0 s, (**b**) at t=300 s.

The results of the modelling of electric potential can be seen in Figure 12 and Figure 13. It can be seen that almost the whole voltage drops in the plasma-gas layer. This was expected from the experimental data. This result allowed us to assume that the plasma-gas layer could be considered as a special electrochemical cell, where the interface between the plasma-gas layer and the electrolyte acts as cathode.

**Figure 14:**
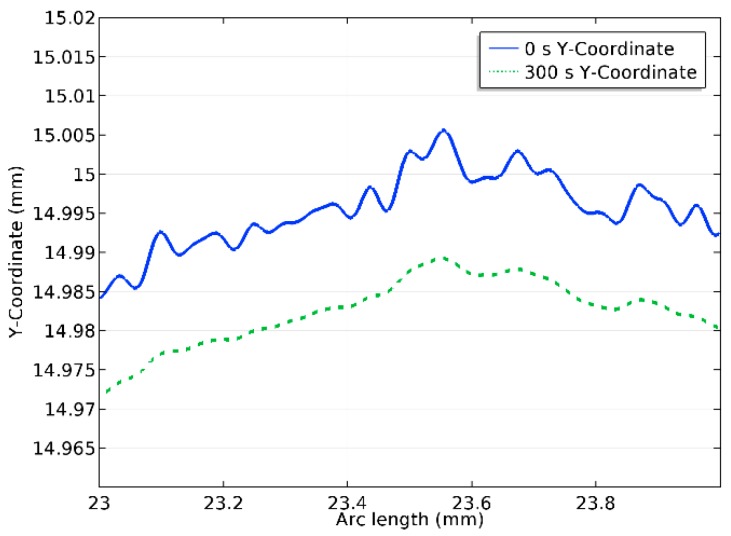
Detailed view of surface profile at 0 s and 300 s.

**Figure 15:**
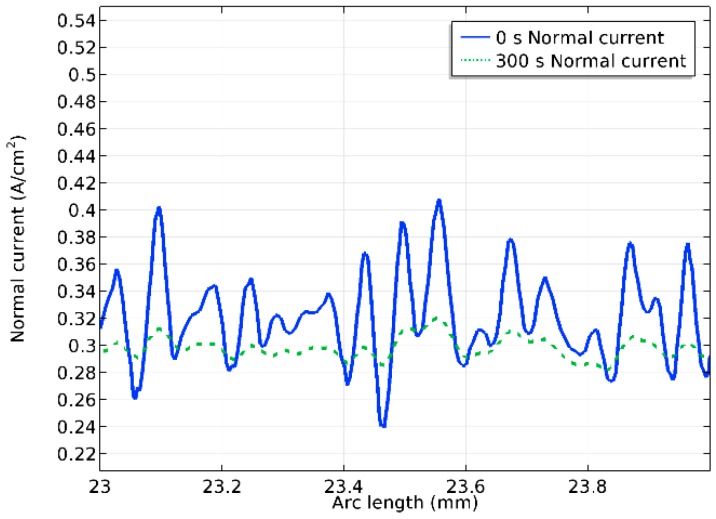
Detailed view of normal current density at 0 s and 300 s.

Figure 14 shows the surface profile before and after 300 s polishing. In Figure 15, the normal current density on the anode surface at the beginning of the process and after 300 s polishing is provided. It can be seen that despite the fact that the overall shape of the surface is retained, the peaks were visibly removed. 

Comparing Figure 15 and Figure 14, it can be concluded that the normal current density is mainly influenced by the shape of the surface. This leads to higher current densities on the surface profile peaks and lower ones in the cavities. Because of the electrochemical mechanism of the process, a higher current density on the peaks, and consequentially, a higher removal rate than in the cavities, leads to a polishing effect on the workpiece surface. In Figure 15, it can also be seen that the current density in the deeper cavities increases with the processing time; this can be explained by the decrease in peak heights, and therefore, a more even current distribution on the surface.

**Figure 16:**
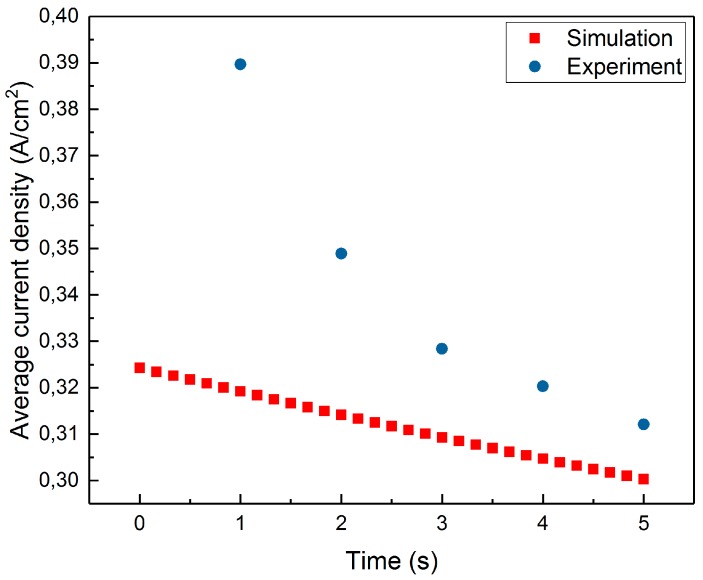
Average current density in model (red) and experiment from Rajput (blue) [13].

Figure 16 presents a comparison of the average current density in the model and in the experiment of Rajput et al. [13].

The time average current density in the model is 0.312 A/cm^2^, compared to 0.340 A/cm^2^ in the experiment from Rajput [13]. It can be seen that the average current density in the model is systematically lower than that in the experiment. This can be explained by the fact that the thermodynamic effects are not taken into account in the model. In the real process of PeP, a large amount of the current is used to heat the anode and electrolyte and to evaporate the electrolyte. Because at the beginning of the process, there is no plasma-gas layer, the current density is maximal. Presumably, a lot of energy is used at the beginning of the process to form the plasma-gas layer. Then, part of the energy is required to stabilise it. In the model, plasma exists from the beginning and remains stable throughout the simulation time. So, no energy is used in the formation and stabilisation of the plasma-gas layer. 

To analyse the polishing effect, the roughness parameter Ra was calculated. The equation for Ra was developed based on the following formula [38]:

(8) $$Ra=\frac{1}{l}\int_{0}^{l}\left|h\left(x\right)\right|dx,$$

where:

l is the evaluation length and h(x) represents deviations from the mean line at position x.

(9) $$h\left(x\right)=\left|y-\bar{y}\right|,$$

To calculate this in COMSOL, the following component couplings were used: intop1 - integration over boundaries 13 and 14, and aveop1 – average over the sample boundaries. 

Because the workpiece is a disc, it is necessary to make some changes to this equation. First of all, *x* in this case is changed to the radius, *r,* of the disc. So, the deviations from the mean line were calculated using the following equation:

(10) $$h\left(x\right)=\left|r-\bar{r}\right|,$$

where $\bar{r}$ is an overage radius, calculated with aveop1. 

Then, for the disc, l is the circumference. The following equation was used:

(11) $$l=2\pi \bar{r},$$

Applying everything to equation (8):

(12) $$Ra=\frac{1}{2\pi \bar{r}}\int \left|r-\bar{r}\right|dl,$$

**Figure 17:**
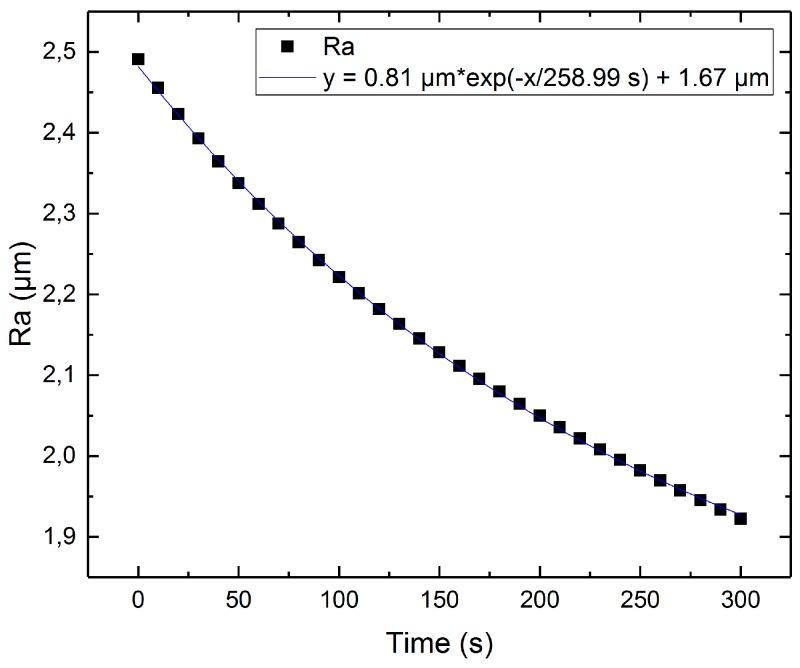
Selected results for Ra as function of time with fit curve.

The results of this calculation are presented in Figure 17. It can be seen that the roughness decreases according to exponential decay (1) from Mukaeva [14]. According to this equation, the minimal achievable roughness Ra in this model has a value of 1.67 µm.

## 3. Inline Metrology in Plasma Electrolytic Polishing

Inline metrology systems are important to ensure product quality control during processing. In the case of plasma polishing, the most important parameter of the final product is the surface roughness. To ensure the stability of the polishing process, it is necessary to control the current density and temperature.

According to the existing literature, common value of average current density range from 0.1 A/cm^2^ to 0.5 A/cm^2^ [1,2,3,5,7,16,34,40]. Thus, knowing the initial sample area and monitoring the current during processing, the process can be controlled. If the current density values are too large or too small, this may mean that the process is not stable; it could indicate, for example, that the plasma-gas layer is unstable or that it has collapsed. In this case, the end result may exceed the requirements. In addition, based on the experimental data, it can be assumed that the current can be analysed to gain information about the surface roughness.

Temperature control is important because the temperature directly affects the formation of the plasma-gas layer, and thus, the current during the process. At higher temperatures, the plasma-gas layer thickness increases, which leads to a decrease in current density. Also, if the temperature is too high, it can lead to the destruction of the chemical components of the electrolyte and/or to their evaporation.

**Figure 18:**
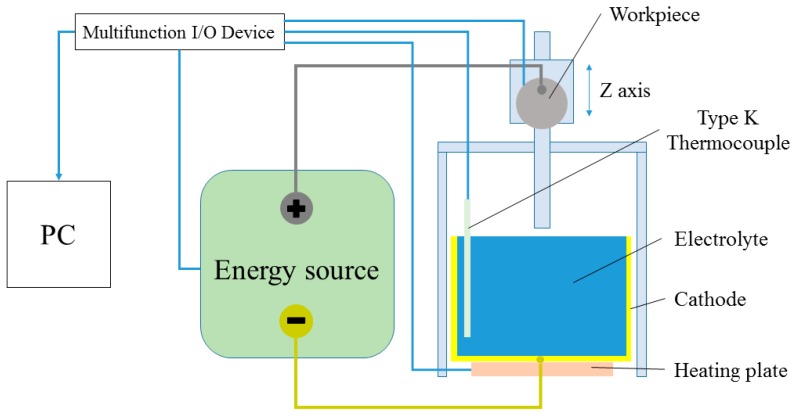
Scheme of the PeP prototype system.

A PeP prototype lab system was developed at Chemnitz University of Technology. A summary of the system can be seen in Figure 18. The setup has one axis in the z-direction. Different clamping systems for the sample can be mounted. The selected power supply Keysight N8762A makes it possible to set the voltage up to 600 V and the current up to 8.5 A, and includes a built-in current measurement system. 

The temperature was measured by thermocoupling using a multifunction I/O device NI USB-6215. The whole system was controlled by PC with controlling software which was developed in LabVIEW. Before starting, a heating plate heated the electrolyte to a pre-set temperature. 

A disc with a 30 mm diameter was selected as the sample for experiment. Each disc had a bore with a 3.8 mm diameter for mounting. A simple hook was used as a sample holder. The discs were made with different initial roughnesses on both sides. All samples were immersed at the same depth. The measured current values were corrected for the current value obtained when the holder hook was immersed without a sample. The initial parameters for experiment are given in Table 5.

The experiment was undertaken to measure changes in mass and roughness. Surface roughness was measured with 3D Laserscanning-Microscope Keyence VK-9700. Sa and Ra roughnesses were chosen as the main parameters for the measurement. The mass of the samples was measured with precision balances Sartorius ME36S. 

Sa and Ra roughnesses were measured before and after polishing at different positions on the sample surface. Two positions were chosen for measurement: one on the top and one on the bottom of the sample on each side. 

### Experimental Results

**Figure 19:**
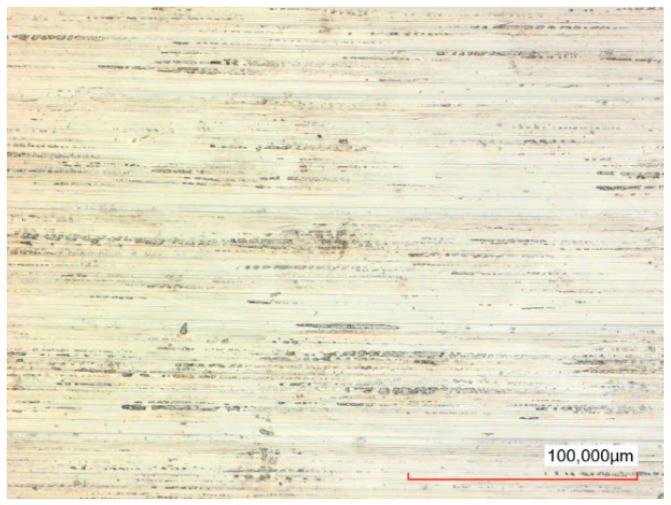
Sample surface before polishing, Ra = 0.080 µm, Sa = 0.124 µm.

**Figure 20:**
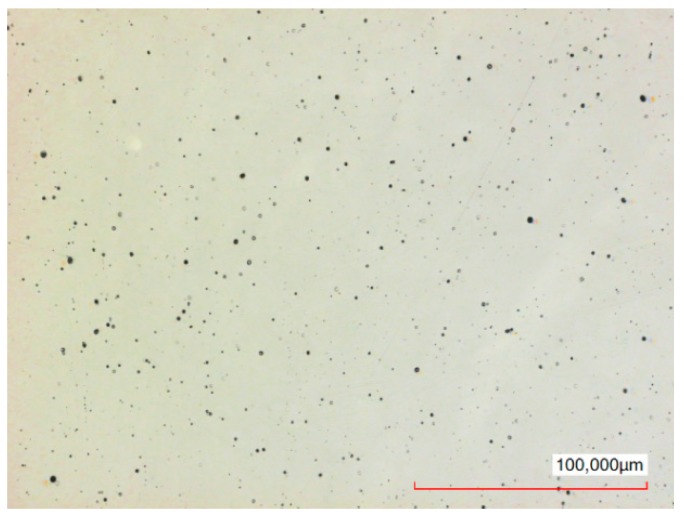
Sample surface after 5 min polishing, Ra = 0.023 µm, Sa = 0.045 µm.

The typical surface of the disc before and after polishing can be seen in Figure 19 and Figure 20 respectively. In Figure 19, the pattern of the surface after turning can be seen. In Figure 20, this pattern is not visually observed on the polished surface. At the same time, dark spots can be observed on the entire polished surface. Some of them are peaks of up to 5 µm. Presumably, these may be undissolved inclusions of carbon or other elements from the composition of the steel. 

**Figure 21:**
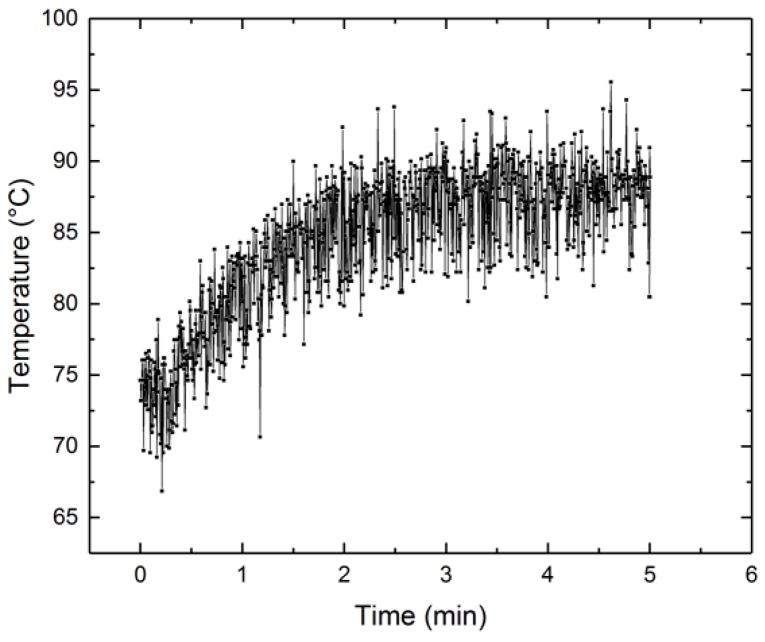
Monitored temperature as a function of time.

Figure 21 shows an example of a monitored temperature of the electrolyte as a function of time. This figure demonstrates a typical increase in temperature during polishing of a single sample. In this example, it increases from about 75 °C to approximately 85 °C after 100 s, and then increases only slightly more in the last 200 s of the process.

**Figure 22:**
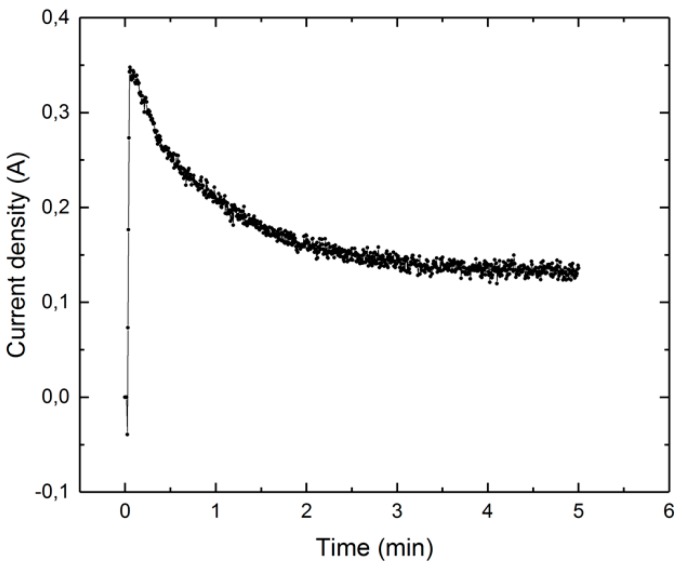
Current density as function of time.

Figure 22 displays an example of current density as function of time. This figure shows a typical decrease in current density during the polishing of a single sample. There are two possible reasons for the observed decrease; firstly, the decrease in current density can be explained by the increase in temperature [14]. At a higher temperature, the thickness of the plasma-gas layer increases, leading to a decrease in current. An alternative possible reason may be the decrease in roughness. Because of the roughness, the current density distribution is not even. The current density is focused mainly on the peaks, so it is higher there. Consequently, the removal rate for the peaks will be also higher, and they will be removed more rapidly. This leads to a fast decrease in the heights of these peaks and, because of this, in roughness. The decrease in the heights of the peaks leads to more even distribution of the current density and a decrease in the current density over time.

**Figure 23:**
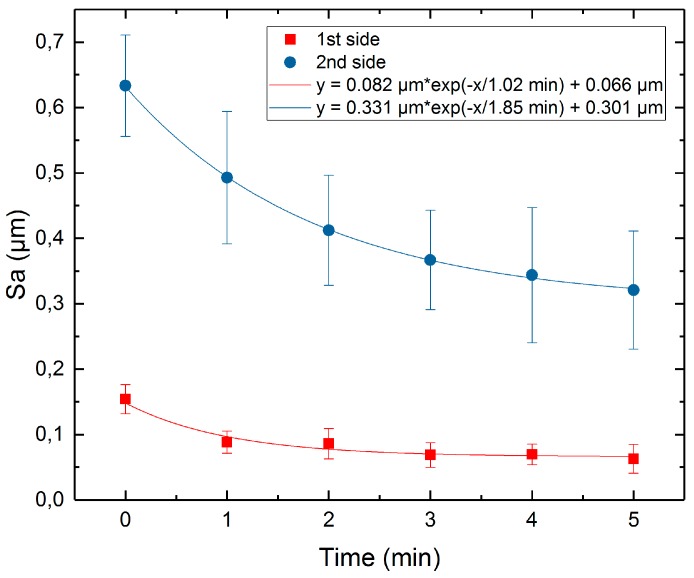
Surface roughness *Sa* as function of time.

**Figure 24:**
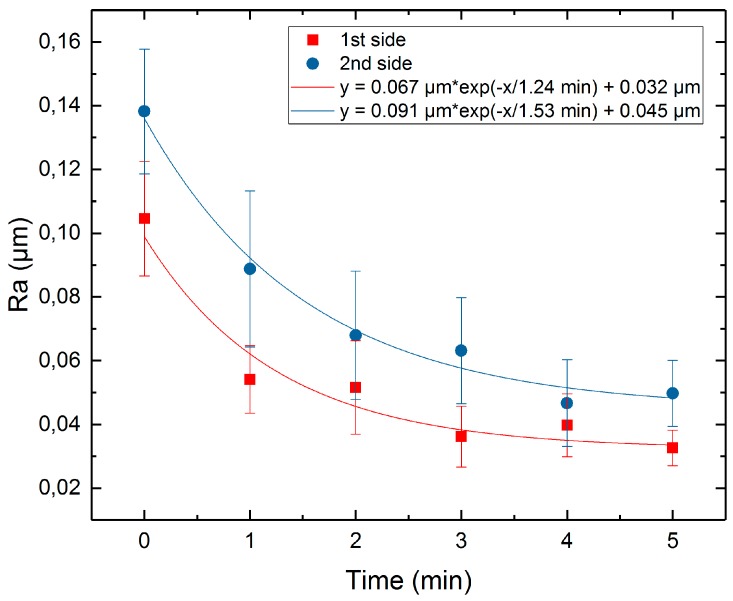
Surface roughness *Ra* as function of time.

Figure 23 shows the average surface roughness, Sa, at both sides of the samples as a function of time with the exponential decay fit function (1). The results for average Ra roughness are presented in Figure 24. 

It can be seen that the exponential decay fit works for Sa roughness, as well as for Ra. The minimum achievable roughness, Sa, for initial roughnesses of 0.15 µm and 0.63 µm, are 0.066 µm and 0.301 µm, respectively. The minimum achievable roughness, Ra, for initial roughnesses of 0.10 µm and 0.14 µm, are 0.032 µm and 0.045 µm, respectively.

Also, the roughness along the sample height was studied. The results are provided in Table 6. On both sample sides, roughnesses Ra and Sa change differently, depending on the position along the height. It can be seen that the decrease in roughness for the bottom of the samples is bigger; this can be explained by the continuous gas formation which occurred during the process. The plasma-gas layer formed in the first seconds of the process and remained stable until the end of processing, mainly due to the evaporation of the electrolyte [10,14,16,24,25,26]. However, the gas moved up along the sample height; this led to an increase in the thickness of the plasma-gas layer from the lowest point of the sample to the highest. Because of this, the current density increased from the top to the bottom of the sample. Taking into account the assumption that PeP is mainly an electrochemical process, it may be concluded that a higher current leads to a higher removal rate. Based on the data obtained in the simulation of the PeP process, we can conclude that the removal rate for the peaks is higher; this also leads to a decrease in the roughness. Thus, a higher current density at the bottom of the sample leads to a faster reduction of roughness.

**Figure 25:**
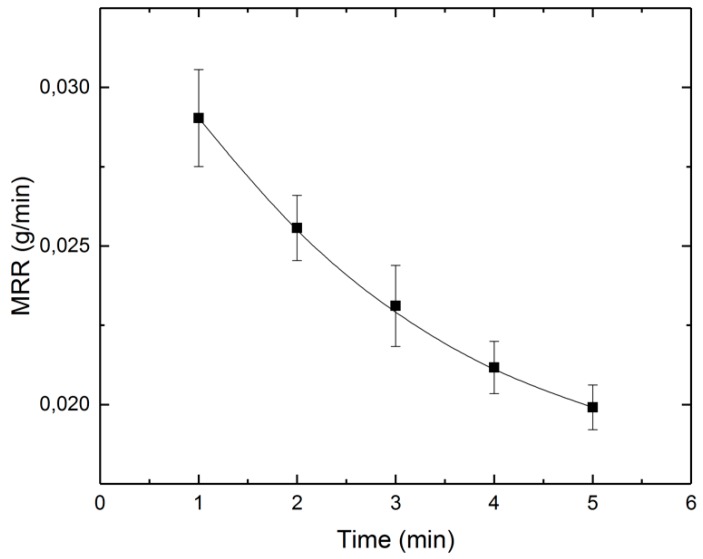
Average MRR as function of time.

The samples were weighed before and after polishing to calculate the material removal rate and current efficiency. The result of the material removal rate (MRR) calculation is provided in Figure 25, in which the average MRR decrease for the longer processing times are given. This may be due to the decrease in current over time. This leads to a decrease in charge, and therefore, to less removal.

The current efficiency was calculated to evaluate the process, and based on the assumption that the PeP process can be considered an electrochemical one. The calculation was based on equations (13) - (15). The parameters used for the calculation can be seen in Table 7.

(13) $$m_{spec}=\frac{1}{F}\overset{n}{\underset{i=1}{\sum }}\frac{c_{i}\cdot M_{i}}{z_{i}},$$

where:

$c_{i}$ is the mass fraction, $M_{i}$ is the Molar Mass, and $z_{i}$ is the valence

(14) $$m_{eff}=\frac{m_{rem}}{Q},$$

where:

$m_{rem}$ is the real mass removal and Q is the exchanged electric charge

(15) $$\eta =\frac{m_{eff}}{m_{spec}},$$

**Figure 26:**
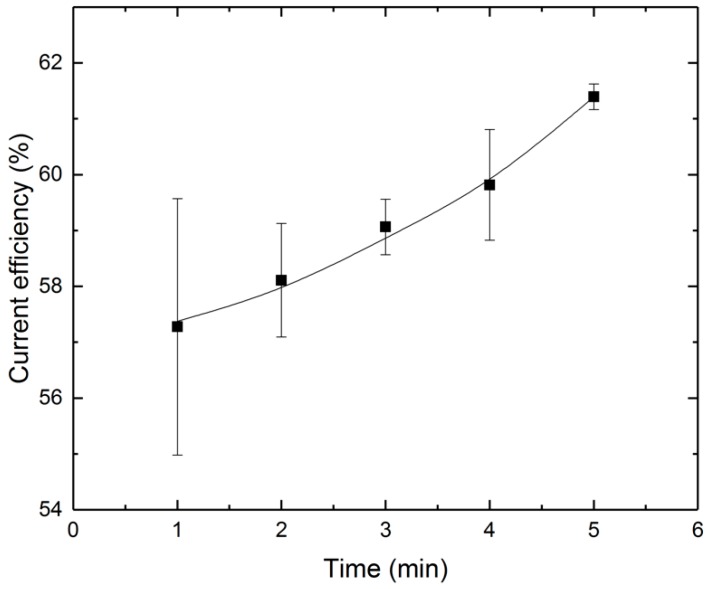
Current efficiency as function of time.

The results of the calculation can be seen in Figure 26. This figure shows the current efficiency of a process for five different processing times. The processing time for an average current efficiency is around 59%. This value is similar for electrochemical machining, but for the same current density values of ECM, current efficiency can be lower due to passivation. As mentioned above, Kellogg [23] and Parfenov et al. [34] made the assumption that when the plasma-gas layer is stable, oxygen formation no longer occurs on the workpiece surface, but rather that oxygen formation still takes place but on the interface between plasma-gas layer and electrolyte. 

It also can be seen in Figure 26 that the current efficiency increases over time. This can be explained by the change in current density over time, as shown in Figure 22. It can be assumed that a decrease in current leads to a lower level oxygen formation, and therefore to lower passivation. 

## 4. Conclusions

This paper provides a review of the literature on the possible mechanisms of the plasma-gas layer conductivity, and on the possible material removal mechanisms during plasma electrolytic polishing. The most popular theories about conductivity in the plasma-gas layer are based on the assumption that the plasma-gas layer is a highly-ionized medium in which different ions and electrons provide conductivity. The two most popular theories about the removal mechanism are electrochemical removal and removal because of melting. 

Based on our simulation and experimental results, the following conclusions may be drawn:

- The main voltage drop in PeP occurs in the plasma-gas layer. The current distribution inside plasma-gas layer is determined by the workpiece surface. Thus, a higher current density on the peaks leads to a faster removal of the peaks and to a reduction of roughness.
- PeP can be simulated as an electrochemical machining process. Moreover, future simulations can be undertaken considering only the plasma-gas layer where the interface with the electrolyte acts as a cathode and the workpiece surface as the anode.
- PeP processing time and Ra roughness can be predicted with the simulation.
- Changes in roughness depend on the position along the sample height.
- Sa roughness can be fitted with exponential decay fit, as well as with Ra roughness.
- The changes in roughness for both Sa and Ra depend on the position along the sample’s height. The decrease in roughness for the bottom of the sample is bigger.
- The current efficiency of PeP is comparable to that of ECM, but the current density is lower.
- Passivation in PeP and oxygen formation should be different from a typical electrochemical process because of the plasma-gas layer.

Future experiments comparing PeP and ECM with the same electrolyte and same current density value are planned, in order to compare the resulting current efficiencies and surface topologies. Furthermore, it will be important to conduct a study of the residual stresses after applying PeP in the future. 

## Figures and Tables

**Table 1:** Experimental parameters [14].

Parameter	Value
Voltage	250 V, 300 V, 350 V
Electrolyte	Ammonium sulfate
Electrolyte concentration (wt %)	5%
Electrolyte temperature	70 °C, 80 °C, 90 °C
Samples material	Stainless steel (1.4021)
Initial roughness (Ra)	0.63 µm, 0.32 µm

**Table 2:** Parameters for Spatial Frequencies method.

Parameter	Description	Value
N	Spatial frequency resolution	2000
b	Spectral exponent	0.5
A	Scale parameter in y coordinate	0.001
s	Phase coefficient	from 0 to 1
g1	Gaussian random function	
u1	Uniform random function	

**Table 3:** Simulation parameters.

Parameter	Value
Voltage	200 V
Anode conductivity	1.38×10^7^ mS/cm
Electrolyte conductivity	120 mS/cm
Plasma-gas layer conductivity	2.55×10^−2^ mS/cm
Plasma-gas layer thickness	0.15 mm
Anode relative permittivity	1
Electrolyte relative permittivity	55
Plasma-gas layer relative permittivity	1

**Table 4:** Parameters for mesh.

Parameter	Electrolyte and Plasma-Gas Layer	Workpiece
Maximum element size	20 mm	20 mm
Minimum element size	0.005 mm	0.005 mm
Maximum element growth rate	1.5	1.2
Curvature factor	0.2	0.2
Resolution of narrow regions	1	1

**Table 5:** Initial parameters.

Parameter	Value
Voltage	250 V
Pre-set temperature of electrolyte	75 °C
Electrolyte salt	Ammonium sulphate
Electrolyte salt concentration	5% of mass
Samples material	steel 1.4301 (AISI 304)
Initial roughness Sa	(0.15 ± 0.02) µm (0.63 ± 0.08) µm
Initial roughness Ra	(0.10 ± 0.02) µm (0.14 ± 0.02) µm

**Table 6:** *Sa* and *Ra* minimum achievable roughness.

Parameter	Initial Roughness	Minimum Achievable Roughness	
		Top	Bottom
Sa	0.15 µm	0.083 µm	0.045 µm
	0.63 µm	0.414 µm	0.221 µm
Ra	0.10 µm	0.033 µm	0.028 µm
	0.14 µm	0.055 µm	0.032 µm

**Table 7:** Parameters for stainless steel 1.4301.

Chemical Element	Fe	Cr	Ni	N	Mn	Si	C
Mass fraction c in %	68.8	18	10	0.1	2	1	0.1
Valence z	3	6	2	3	2	4	4
Molar Mass M in g/mol	55.85	51.996	58.7	14.007	54.94	28.09	12.01
